# Acute lung injury in mechanically ventilated patients with epidermal necrolysis: an exposed-unexposed retrospective cohort study

**DOI:** 10.1093/burnst/tkaa041

**Published:** 2020-12-08

**Authors:** S Lalevée, J Catano, S Ingen-Housz-Oro, M Surenaud, J Tran Van Nhieu, F Schlemmer, I Bendib, A Mekontso-Dessap, S Hue, N de Prost

**Affiliations:** 1 Service de dermatologie, AP-HP, hôpital Henri Mondor, Créteil, France; 2 Université Paris Est Créteil, INSERM U955, IMRB, F-94010 Créteil, France; 3 Service de Médecine Intensive Réanimation, Hôpitaux Universitaires Henri Mondor, Assistance Publique-Hôpitaux de Paris, Créteil Cedex 94010, France; 4 EA7379 EpidermE, UPEC, Créteil, France; 5 Centre de référence des dermatoses bulleuses toxiques et toxidermies graves TOXIBUL, Créteil, France; 6 Service d’Anatomopathologie, AP-HP, hôpital Henri Mondor, Créteil, France; 7 Unité de Pneumologie, Service de Médecine Intensive Réanimation, Hôpitaux Universitaires Henri Mondor, Assistance Publique-Hôpitaux de Paris, Créteil Cedex 94010, France; 8 Groupe de Recherche Clinique CARMAS, Faculté de Santé de Créteil, Université Paris Est Créteil, Créteil Cedex 94010, France; 9 AP-HP, Département d'Hématologie et d'Immunologie biologiques, Groupe hospitalo-universitaire Chenevier Mondor, Assistance Publique-Hôpitaux de Paris F-94010 Créteil, France

Highlights

- At the acute phase of epidermal necrolysis, patients requiring mechanical ventilation had more alveolar macrophages and higher alveolar concentrations of IL-13 than age- and sex-matched patients with the acute respiratory distress syndrome
- Patients with epidermal necrolysis also showed longer duration of mechanical ventilation and more frequent ventilator-acquired pneumonia episodes

To the Editor,

During the acute phase of epidermal necrolysis (EN, including Stevens-Johnson syndrome and toxic epidermal necrolysis), mucosal lesions involving the tracheo-bronchial tract have been reported [1, 2] and approximately one in four EN patients develop acute respiratory failure and require endotracheal intubation and mechanical ventilation (MV) support [3]. Asymptomatic respiratory sequelae may be detected on pulmonary function tests after the acute phase in as much as 50% of EN patients, possibly related to pulmonary lesions that occurred at the acute phase [4]. EN is drug-induced in 85% of cases, and considered a T-cell mediated, type IV hypersensitivity disorder with predominant activation of CD8^+^ T lymphocytes, monocytes/macrophages, and natural killer cells. Yet, the pathophysiology of respiratory system involvement during the acute phase of EN is not well understood. The current study aimed at exploring the clinical and biological characteristics of acute lung injury during the acute phase of EN in mechanically ventilated patients.

**Table 1:** Characteristics of patients with epidermal necrolysis and acute respiratory distress syndrome

	EN (n = 15)	ARDS (n = 15)	*P* value
**Characteristics at ICU admission**			
Age	45 (25–57)	47 (35–57)	0.705
Gender, male	10 (67)	10 (67)	>0.99
Main comorbidities			-
COPD	1 (7)	2 (13)	
Tobacco smoking	3 (20)	4 (27)	
Diabetes	1 (7)	4 (27)	
Obesity	0 (0)	2 (13)	
Chronic hemodialysis	1 (1)	0 (0)	
Chronic heart failure	0 (0)	1 (1)	
Immunosuppression	0 (0)	0 (0)	
SAPS II score	27 (19–45)	36 (29–55)	0.108
SOFA	4 (2–6)	8 (7–11)	**<0.001**
PaO_2_/FiO_2_ ratio, mmHg	286 (248–412)	130 (82–198)	**<0.0001**
Detached BSA, %	13 (0–25)	-	-
Median SCORTEN	2 (1–2)	-	-
**Characteristics during ICU stay**			
Maximal detached BSA, %	30 (15–60)	-	-
EN-specific tracheal/bronchial lesions	9 (60)	-	-
Shock	11 (73)	9 (60)	0.700
Renal replacement therapy	2 (13)	8 (53)	**0.050**
Lowest PaO_2_/FiO_2_ ratio, mmHg	243 (98–300)	103 (62–178)	**0.019**
Ventilator-associated pneumonia	9 (60)	3 (20)	0.060
Duration of mechanical ventilation, days	16 (11–22)	9 (3–22)	0.054
Duration of ICU stay, days	21 (14–24)	10 (7–32)	0.091
Hospital mortality	2 (13)	5 (33)	0.399
ICU admission—BAL^a^, days	2 (1–5)	1 (1–2)	**0.048**
**BAL fluid cytology**			
Cellularity (10^3^ cells/mL)	440 (130–780)	450 (104–1043)	0.771
Macrophages, %	74 (50–80)	34 (18–53)	**0.001**
Neutrophils, %	18 (6–46)	53 (40–75)	**0.002**
Lymphocytes, %	3 (2–10)	6 (2–10)	0.559
Eosinophils, %	0 (0–2)	0 (0–0)	-

Qualitative variables are shown as number (percentages); continuous variables are shown as median (quartile 1–quartile 3); for qualitative variables, *p* values come from the Chi^2^ or Fisher tests, as appropriate; for continuous variables, *p* values come from the Mann–Whitney test.

^a^Time lag between intensive care unit admission and broncho-alveolar lavage sampling; *ARDS* acute respiratory distress syndrome, *COPD* chronic obstructive pulmonary disease, *EN* Epidermal necrolysis, *SAPS II* Simplified Acute Physiology Score II, *BSA* body surface area, *ICU* intensive care unit; **Bolded** values are significant at the *p* < 0.05 level

**Figure 1.**
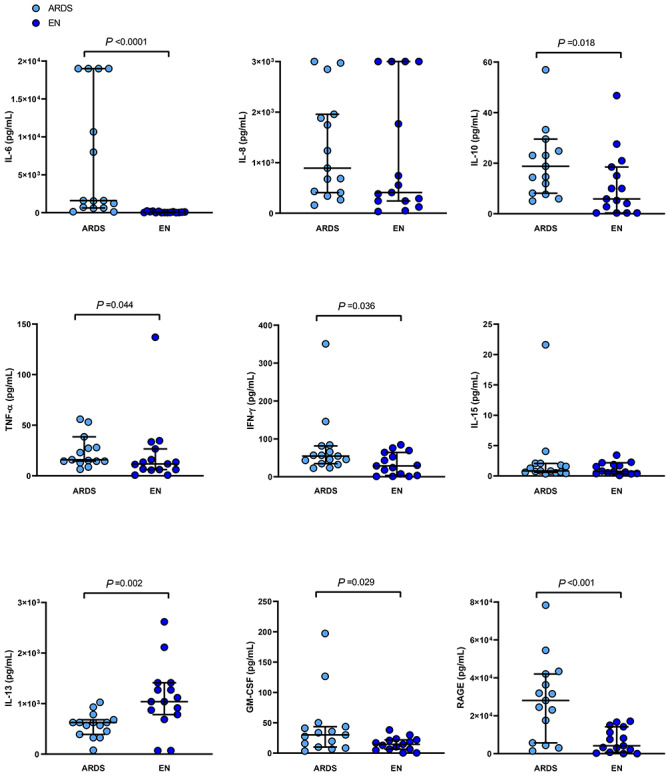
Broncho-alveolar lavage fluid concentrations of cytokines and epithelial injury biomarkers in patients with epidermal necrolysis (EN, navy blue) and the acute respiratory distress syndrome (ARDS, sky blue). *P* values come from the Mann–Whitney test. Horizontal lines indicate median values and interquartile ranges. *IL* interleukin, *TNF-α* tumor necrosis factor α, *IFN-*γ interferon γ, *GM-CSF* granulocyte-macrophage colonystimulating factor, *RAGE* receptor for advanced glycation end products

This is a retrospective monocentric cohort study including patients with EN admitted in the intensive care unit (ICU) of the French National Reference Center for EN during 2011 and 2015 who required endotracheal intubation for acute respiratory failure. This observational cohort study was approved by the institutional ethics committee (French Intensive Care Society, #CE SRLF 19–29). Patients received information during hospital stay that data abstracted from their medical charts could be used for research purposes.

EN patients were age- and sex-matched with a historical control cohort of patients with pneumonia-associated acute respiratory distress syndrome (ARDS) [5]. As standard of care, all patients underwent a bronchoscopy with broncho-alveolar lavage (BAL) sampling within the first week of ICU admission. During a standard flexible bronchoscopy, four aliquots of normal saline (50 mL each) were instilled through the bronchoscope within the selected bronchopulmonary segment and retrieved using a negative suction pressure. BAL fluid cytology was performed by direct microscopy after centrifuging broncho-alveolar lavage fluid samples (12 000 revolutions for 10 min) and dying under the May-Grünwald-Giemsa staining. Total (quantified in cells/mL) and differential (*i.e.* percent of neutrophils, macrophages and lymphocytes) cell counts were measured as recommended [6]. Cytokines (tumor necrosis factor (TNF)-α, interferon (IFN)-γ, interferon- γ-induced protein 10 (IP-10), interleukin (IL)-8, IL-10, IL-6, IL-13, IL-15, granulocyte-macrophage colony-stimulating factor (GM-CSF)) and receptor for advanced glycation end products (RAGE) levels were quantified in BAL fluid by Luminex technology (Human Magnetic Luminex assay, R&D). Comparisons between EN and ARDS were performed using the Chi^2^ or Fisher tests for qualitative variables and the Mann–Whitney test for continuous variables. Ventilator-associated pneumonia was defined according to the presence of clinical findings suggesting infection (new onset of fever, purulent sputum, leucocytosis, decline in oxygenation) along with a radiographic infiltrate that is new or progressive and a positive distal quantitative sample [7].

Fifteen EN were matched with 15 ARDS patients (Table 1). EN patients presented with a median SCORTEN [8] of 2 and a median maximal detached skin surface of 30% of body surface area. Nine (60%) EN patients displayed bronchoscopy findings consistent with specific EN-related tracheo-bronchial lesions. As compared with ARDS patients, EN patients showed significantly higher PaO_2_/FiO_2_ ratios both at ICU admission and during ICU stay and lower SOFA scores at admission. Yet, EN patients had longer durations of mechanical ventilation than ARDS patients and underwent more frequent ventilator-acquired pneumonia. Total BAL cell counts were not significantly different between EN and ARDS patients (Table 1). However, BAL differential cell counts revealed significantly more macrophages and less neutrophils in EN than in ARDS patients. The percentage of lymphocytes did not differ between EN and ARDS. Patients with ARDS displayed significantly higher BAL fluid levels of cytokines involved in innate immunity than EN patients, including IL-6, IL-10, TNF-α, IFN-γ, GM-CSF, as well as higher levels of RAGE, reflecting more severe alveolar epithelial injury. In contrast, BAL fluid levels of IL-13, a Th2 cytokine, were significantly higher in EN than in ARDS patients (Figure 1). The BAL fluid levels of these biomarkers did not differ according to the presence of macroscopic tracheo-bronchial lesions on bronchoscopy or not (data not shown).

This exploratory study showed different profiles of pulmonary inflammation in EN patients with acute respiratory failure requiring mechanical ventilation support than in age- and sex-matched patients with ARDS, the most severe form of acute hypoxemic respiratory failure. Of note, EN patients included in the current cohort did not all exhibit specific tracheo-bronchial lesions, confirming that acute respiratory failure may occur in EN even in the absence of such mucosal involvement. While EN patients seemed to have less severe acute respiratory failure upon ICU admission than others, these had a more complicated course with longer mechanical ventilation duration, suggesting that the mechanisms involved in acute lung injury may be different. Indeed, EN patients showed a different lung inflammation profile, with a majority of macrophages while ARDS patients showed, as expected, an intense lung neutrophil infiltration [9], consistent with higher levels of IL-6, TNF-α, IFN-γ, GM-CSF. In the context of injury and exposure to inflammatory mediators, CD8^+^ T cells could release type 2 cytokines [10]. Increased levels of IL-13 in EN suggest such mechanism involving an immune response prone to tissue repair.

Our study has some limitations including the small number of patients included, its monocentric design, limiting the generalizability of the findings, and its retrospective nature, with inherently associated bias. Yet, it also has some strengths, including mainly the fact that we included a cohort of age- and sex-matched patients with ARDS.

In conclusion, as compared with ARDS, the pulmonary disease at the acute phase of EN was characterized by a different profile, with more alveolar macrophages and higher levels of IL-13. Whether these features are involved in the pathogenesis of long-term sequelae of EN remains to be determined.

## Declarations

### Ethics approval and consent to participate

This observational cohort study was approved by the institutional ethics committee (French Intensive Care Society, #CE SRLF 19–29). Patients received information during hospital stay that data abstracted from their medical charts could be used for research purposes.

## Consent for publication

Not applicable.

## Availability of data and materials

The dataset used during the current study is available from the corresponding author upon reasonable request.

## Competing interests

All authors declare no competing interest for this work.
